# Components determining the slowness of information processing in parkinson’s disease

**DOI:** 10.1002/brb3.2031

**Published:** 2021-01-15

**Authors:** Aida Arroyo, José A. Periáñez, Marcos Ríos‐Lago, Genny Lubrini, Jorge Andreo, Julián Benito‐León, Elan D. Louis, Juan Pablo Romero

**Affiliations:** ^1^ Facultad de Ciencias Experimentales Universidad Francisco de Vitoria Madrid Spain; ^2^ Experimental Psychology Department Universidad Complutense de Madrid Madrid Spain; ^3^ Basic Psychology II Department UNED, Madrid 28040, Spain; Brain Damage Unit Hospital Beata María Ana Madrid Spain; ^4^ Department of Neurology University Hospital “12 de Octubre”, Madrid, Spain; Centro de Investigación Biomédica en Red sobre Enfermedades Neurodegenerativas (CIBERNED), Spain; Department of Medicine Complutense University Madrid Spain; ^5^ Department of Neurology and Neurotherapeutics at UT Southwestern Medical Center; ^6^ Facultad de Ciencias Experimentales Universidad Francisco de Vitoria, Madrid 28223, Spain; Brain Damage Unit Hospital Beata María Ana Madrid Spain

**Keywords:** Cognition, Human Information Processing, Parkinson´s disease, Reaction Time

## Abstract

**Introduction:**

Bradyphrenia is a key cognitive feature in Parkinson's disease (PD). There is no consensus on whether information processing speed is impaired or not beyond motor performance.

**Objective:**

This study aims to explore which perceptual, motor, or cognitive components of information processing are involved in the slowdown affecting cognitive performance.

**Methods:**

The study included 48 patients with PD (age: 63, 3 ± 8, 18; HY I‐III; UPDRS 15,46 ± 7,76) and 53 healthy controls (age: 60,09 ± 12,83). Five reaction time (RT) tasks were administered to all participants. The average RT in each of the tasks and the percentage of correct answers were measured. Patients with PD were in "ON state" at the time of the evaluation. Perceptual, motor, and cognitive components were isolated by means of a series of ANCOVAs.

**Results:**

As expected, the motor component was slowed down in patients with PD. Moreover, while patients with PD showed slower RT than controls in all tasks, differences between groups did not exponentially increase with the increasing task complexity. ANCOVA analyses also revealed that the perceptual and sustained alert component resulted to be slowed down, with no differences being found in any of the remaining isolated cognitive components (i.e., response strategy‐inhibition, decisional, visual search, or interference control).

**Conclusions:**

The results revealed that slowness of information processing in PD was mainly associated with an impaired processing speed of the motor and perceptual‐alertness components analyzed. The results may help designing new neurorehabilitation strategies, focusing on the improvement of perceptual and alertness mechanisms.

## Graphical abstract

The level of cognitive deceleration in PD is determined by a deterioration focused on perceptual and motor components and independently of the task complexity. Alterations in the brain networks involved in alertness to react to stimuli could justify the slowness of information processing in Parkinson´s disease

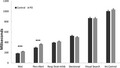

## 1 INTRODUCTION

In Parkinson's Disease (PD) bradyphrenia or slowness in the information processing (SIP) produces alterations in the speed of thought but also increases attentional impairments, cognitive inflexibility, and forgetfulness (Shipley et al., 2002). These symptoms are believed to appear from the initial stages of the disease and even before the motor symptoms occur (Johnson et al., 2016).

Deficits in the information processing speed in Parkinson's disease have been investigated since the 80s through reaction time tasks (RT) in order to establish whether the slowness affects single cognitive mechanisms or whether it is a global impairment across all cognitive mechanisms. Both simple reaction time tasks (SRT), and choice reaction time tasks (CRT), have been investigated in patients with PD with no uniform conclusions (Evarts et al., 1981). On the one hand, different studies indicate that patients are slower in SRT (such as detection and interference tasks), not increasing the difference in CRT (e.g., decision making, visual search, and interference control), which supports the hypothesis of a possible overall deterioration in processing speed (Berry, 1999; Bloxham et al., 1987; Goodrich et al., 1989; Jordan et al., 1992; Sheridan et al., 1987). On the other hand, different authors point to the increase in the difficulty of the task as the main cause of the increase in RT (Cooper et al., 1994; Gauntlett‐Gilbert and Brown, 1998; Jahanshahi et al., 1992; Pullman et al., 1990; Stelmach et al., 1986).

To disentangle whether SIP it is a global or specific deficit, some authors have suggested to explore three different stages in the information processing pathway: sensory input, cognitive mechanisms, and motor output (Malturin, 2013). Firstly, some authors consider that motor slowness (information output) and preprogramming would explain the slower RTs of PD (Smith et al., 1998; Phillips et al., 1999). On the other hand, some other authors consider perceptual deficits as the basis for the SIP in PD (Cooper et al., 1994; Vlagsma et al., 2016). Multiple authors have focused their hypothesis in decision making (Djamshidian et al., 2014), interference control (Verleger et al., 2010), and visual search. (DeGutis et al., 2016) Furthermore, some aspects such as impulsivity (Rossi et al., 2017) and inhibition (Obeso et al., 2011), known to be related to dopaminergic transmission, have also been described as the possible cause of information processing impairments in patients with PD. Nevertheless, previously mentioned studies found no differences between patients with PD and healthy controls in cognitive function (Smith et al., 1998; Phillips et al., 1999).

Given the previously mentioned findings, there have been several intents to isolate single components of information processing through different methodologies. As for the motor components, Sawamoto et al (Sawamoto et al., 2002) concluded that motor deceleration did not explain the higher RT of the PD group by subtracting the motor time from the total RT, thus isolating the motor component from the cognitive component. Using a similar approach, Copper et al (Cooper et al., 1994) subtracted the SRT from the CRT in order to analyze the motor preprogramming reaching the same conclusion. Vlasgma (Vlagsma et al., 2016), using linear regression models, found that patients with PD show mental slowness, which could be separated from motor slowness.

There are no studies in our knowledge to have addressed the isolation of single components of the cognitive processes involved in information processing in PD using computerized RT tasks with gradual increases in complexity. Given this, the present study aimed to clarify the presence of a SIP as a generalized phenomenon or a specific impairment in single components of the information processing pathway in patients with PD. With this purpose, a comprehensive set of computerized RT tasks with an increasing level of cognitive demands was administered to a cohort of patients with PD and a group of healthy controls. Two hypotheses were formulated. First, it was hypothesized that, if the slowness of RT is a generalized phenomenon in PD, differences between healthy controls and patients with PD will arise in all RT tasks regardless of the complexity of the task. Secondly, if it is a global deterioration of information processing, all the components involved in the aforementioned processing (perceptual, cognitive, and motor) will be affected in PD.

## 2 MATERIALS AND METHODS

### 2.1 Participants

A total of 48 patients with PD and 53 healthy controls were recruited in a movement disorders clinic (from November 2017 to March 2018). Both groups were matched in age, sex, and education. The demographic and clinical characteristics of all the participants are shown in Table 1. Exclusion criteria for all participants were: MoCA score < 25 (Nazem et al., 2009), severe dependence (mRS > 3), depression (score ≥ 13 in the Spanish version of the Beck Depression Inventory and/or score ≥ 13 in the rating scale for depression of Hamilton (Lobo et al., 2002; Sanz and Vázquez, 1998)), previous history of neurologic or psychiatric disorder or severe comorbidity, and under 18 years of age. Exclusion criteria were set for patients using advanced therapies for Parkinson's disease (apomorphine pump / duodenal dopamine infusion), epilepsy history or structural alterations in previous imaging studies, and poor response to levodopa or suspicion of atypical parkinsonism's. Inclusion criteria for patients with PD were idiopathic Parkinson's disease (diagnosed according to London brain bank criteria (Hughes et al., 1992)), stage I‐III Hoehn‐Yahr, not having evident motor fluctuations and clinical stability (not having changed the antidopaminergic medication in the last 30 days or antidepressive medication in the last 90 days). Participants with RTs greater than two standard deviations above the mean were excluded. All the participants were informed of the details of the evaluation and signed their consent to participate in this study, in accordance with the declaration of Helsinki. The Ethics Committee of the institution approved the study on 8th July 2016. Code 16/37.

**TABLE 1:** Means (Standard Deviation) of demographic and clinical data from participants

	Healthy Controls	PD	*p* value
*N* (male)	53 (34)	48 (32)	0.627
Age in years	60,09 (12,83)	63,63 (8,81)	0.110
Education in years	13,55 (3,39)	12,48 (3,81)	0.140
HY	‐	HY1 = 12; HY1,5 = 1; HY2 = 24; HY 2,5 = 3; HY 3 = 8. Mo = 2	
UPDRS III	‐	15,46 (7,76)	
PDQ−39	‐	23,63 (14,61)	
Total dopamine	‐	696,90 (424,93)	

Note

HY: Hoehn and Yahr Scale, UPDRS III: Unified Parkinson's Disease Rating Scale III, PDQ‐39: The Parkinson's Disease Questionnaire.

### 2.2 Experimental design and procedures

The study is a case‐control design with cross‐sectional measurement. Evaluation of all the participants was carried out in a single session lasting 60 min after signing the informed consent. The day of the procedure, clinical, and sociodemographic data were collected. Secondly, they were examined with five RT tasks. All patients were examined at least one hour after their last dopaminergic medication dose while in their best ON state and did not refer wearing off symptoms. The tasks were performed using a 15‐inch monitor, controlled by Presentation® software (http://www.neurobs.com). The order of presentation of the task was counterbalanced among the participants. The average RT in each of the tasks and the percentage of correct answers were measured (Table 2). The Unified Parkinson's Disease Rating Scale III (UPDRS III) was assessed for each patient, they were all in "ON state" at the time of the evaluation.

**TABLE 2:** Means (S.D.) of Reaction Times (RT) in milliseconds and percent of correct responses (% correct) for the RT tasks

		Healthy Controls	PD	*p* value
FT	RT ms	187,29 (30,70)	224,35 (66,32)	< 0.001
SRT	RT ms	296,37 (58,80)	371,28 (67,82)	< 0.001
	% correct	97,15 (3,37)	96,41 (7,02)	
SRT‐SART	RT ms	372,68 (59,91)	444,67 (82,44)	< 0.001
	% correct	98,16 (2,22)	97,27 (7,64)	
CRT	RT ms	490,38 (107,15)	540,98 (112,42)	0.023
	% correct	91,04 (10,55)	88,97 (12,48)	
CRT‐ Search	RT ms	825,13 (166,01)	911,34 (238,18)	0.035
	% correct	95,72 (3,95)	93,38 (5,91)	
Target‐Low Int.*	RT ms	738,47 (125,78)	814,21 (213,62)	
	% correct	94,35 (6,52)	92,35 (7,27)	
No‐Target‐ Low Int.*	RT ms	828,54 (192,98)	912,15 (273,70)	
	% correct	97,54 (9,83)	96,61 (5,25)	
No‐Target‐High Int*	RT ms	966,17 (213,24)	1,081,08 (319,69)	
	% correct	95,84 (5,24)	93,55 (7,50)	

Note

*: Conditions of the CRT‐Search task; CRT: Choice Reaction Time task; CRT‐Search: Choice Reaction Time‐Search task; FT: Finger Tapping; Int: Interference; SRT: Simple Reaction Time task; SRT‐SART: Simple Reaction Time‐SART task.

Finger Tapping (FT): The FT task was used as a measure of motor function (Reitan and Wolfson, 1996). This task is very sensitive to the slowing down of responses (Strauss et al., 2006). In studies with patients with PD, it has been used as a measure of motor speed (Bronte‐Stewart et al., 2000). In this task, following the Strauss application norms (Strauss et al., 2006), the participants were instructed to press the spacebar on the keyboard as fast as possible and repeatedly with the index finger. Five 10‐s attempts were performed with the dominant hand. The average time between two consecutive taps in the five trials was the dependent variable.

Simple Reaction Time (SRT): Inspired by the SRT task of the Computerized Information Processing Testing (CTIP) battery (Reicker et al., 2007), this task was used as a measure of simple perception and sustained alertness (Jensen, 2006). In PD, it has been used as a SIP evaluation measure (Jahanshahi et al., 1992). Participants were instructed to press the left mouse button as fast as possible when the stimulus "+" appeared in the center of the screen at a size of 2 cm x 2 cm. The order of appearance was constant for all participants. The task consisted of 50 trials lasting 2–3 min.

Simple Reaction Time‐Sustained Attention to Response Task (SRT‐SART): This task allowed to measure response strategy‐inhibition. Similar tasks have been used previously in PD (Yang et al., 2018). Following the model of Robertson et al. (Robertson et al., 1997), participants had to press the left mouse button when the stimulus (digits 1–9) appeared in the center of the screen and inhibited the response when it appeared as the number "3." The task consisted of 168 Go trials and 21 No / Go trials; the average duration was 4 min. Stimuli varied in size between 12mm and 29mm.

Choice Reaction Time (CRT): This task was used as a measure of visual perceptual decision time, and it is related to the same processes involved in the SRT plus the processing of uncertainty as to which one of the stimulus would appear next, that is, decisional processing. Decision processing has been used with patients with PD as a measure of SIP (Hocherman et al., 2004). Following the model of Chiaravalloty et al. (Chiaravalloti et al., 2003), participants had to press the left mouse button when a square appeared in the center of the screen (4 cm x 4 cm) or press the right button when a circle appeared. The task consists of 80 tests with a duration of approximately 3 min.

Choice Reaction Time‐Search (CRT‐Search): Following the model of Neisser (Neisser, 1964), this task was used to measure visual search. Participants had to press the left mouse button when a "Z" appeared in a sequence of 6 letters or press the right button when it did not appear. Stimuli were classified according to two dimensions: presence/absence of "Z" (target/non‐target stimulus) and the visual characteristics of the rest of the letters in the sequence (rounded or angular, high/low interference). The combination of both gives us four different experimental combinations: Target‐Low interference (e.g., GODZCQ); Target‐High Interference (e.g., VWMZEX); Non‐Target‐Low interference (e.g., CQUGRD); Non‐target‐High Interference (e.g., VXWEIM). The letter "Z" cannot appear in the first or sixth position. The task consisted of 128 trials lasting between 5 and 8 min.

### 2.3 Statistical analysis

Differences between the groups in the demographic variables (sex, age, and education) were determined through the *t* tests or Chi‐square.

A task x group mixed ANOVA was performed, with task as the within‐subject factor (FT, STR, STR‐SART, CRT, and CRT‐Search) and group as the between‐subject factor (PD or healthy control.) In those cases in which the spherical assumption of the linear general model was not met, the epsilon corrector (ε) of Greenhouse–Geisser was applied to the ANOVA, which corrects the degrees of freedom. For multiple comparisons, the level of significance was adopted *p* < .05. Secondly, independent samples *t* tests and ANCOVAs were used to identify those cognitive components contributing to RT performance as detailed below. (1) The presence of information processing slowness associated with a “motor” component was analyzed through independent sample *t* tests comparing the response time in the FT task between groups; (2) the slowness in the processing of the information associated with the “perceptual and sustained alert components “was analyzed by an ANCOVA with the RT in the SRT task as the dependent variable and the response time in the FT task as the covariate. Use of FT as a covariate allows controlling the shared “motor” component with the STR task; (3) the response strategy‐inhibition component was isolated by an ANCOVA in which RT in the SRT‐SART task was used as a dependent variable and RT in the SRT task as the covariate, allowing to control the “motor, perceptual, and sustained alert” components; (4) slowness in the processing associated with the decision time marked by visual perception, hereinafter "decisional", was carried out by means of an ANCOVA with the RT in CRT as the dependent variable and RT in the SRT task as the covariate, since in both tasks they involve the same processes plus the decision process in CRT; (5) presence of slowness associated with the visual search was analyzed by an ANCOVA with the RT in the "non‐target‐low interference" condition of CRT‐Search as the dependent variable and the "target‐low interference" condition as the covariate, being able with this last to control the perceptive, motor, and cognitive processes, excepting the visual search. (6) To measure the “interference control” component, an ANCOVA was performed with the "Non‐Target‐High Interference" condition of the CRT‐Search task as the dependent variable and RT in the "Non‐Target‐Low Interference" condition as the covariate. The conditions "Non‐Target‐High Interference" and "Non‐Target‐Low Interference" were used because they imply the same type of visual search, but different levels of interference caused by distractors. Because the distractors are very different from the target in the "Non‐Target‐Low Interference" condition, the search occurs faster than in the "Non‐Target‐High Interference" condition. The level of significance *p* < .05 was adopted for all analyses.

An analysis of the variance (ANOVA) of repeated measures task x group was carried out, where the intrasubject factor was the percentage of correct responses in the four tasks (STR, STR‐SART, CRT, and CRT‐Search), and the group factor (PD or healthy control). The assumptions of ANOVA and ANCOVA were verified (Appendix A). The significance level was adopted *p* < .05.

Due to the heterogeneity of the PD patient sample, Pearson correlations were performed between the different TR and the total dopamine dose and a Spearman correlation between the different TR and the HY stage. The significance level was adopted *p* < .05. All analyses were performed using SPSS v 19.0.3.

## 3 RESULTS

### 3.1 Demographics

No differences were found between PD and healthy controls in sex (χ2(1) = 0.93; *p* = .627), age (t (92) = −1.642; *p* = .11, and educational level (t (99) = 1.49; *p* = .14).

### 3.2 Reaction time

**FIGURE 1:**
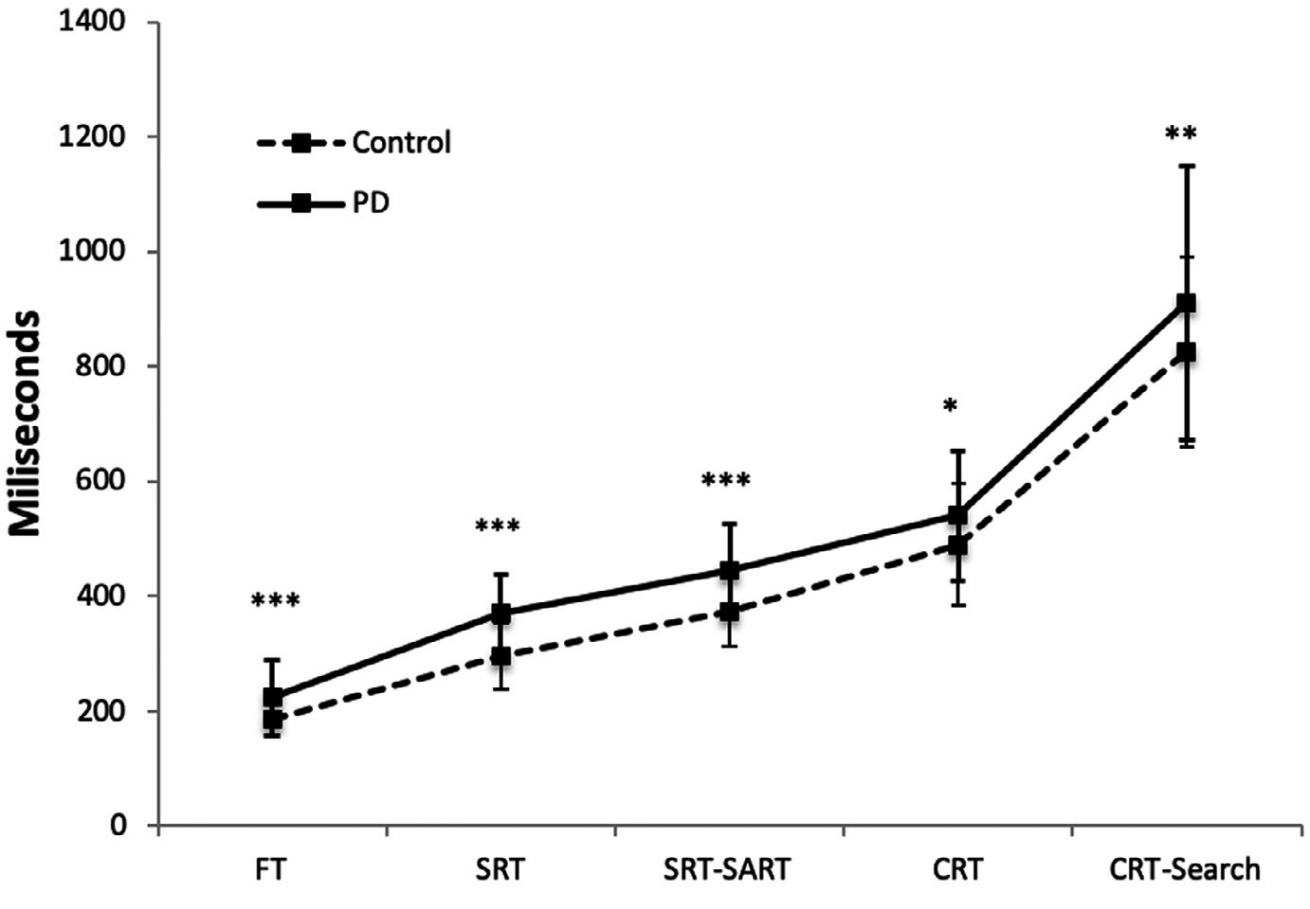
Comparison between reaction times (milliseconds) of healthy controls with patients with PD; FT: Finger tapping, SRT: Simple Reaction Time; SRT‐SART: Simple Reaction Time–Sustained Attention to Response Task; CRT: Choice Reaction Time; CRT‐Search: Choice Reaction Time–Search.*p < .05, **p < .01. ***p < 0,001

ANOVA comparing the RTs in patients with PD and controls revealed a main effect of the group (*F* (1, 98) = 17.38; *p* < .001) showing that patients with PD had slower RTs. There was also a main task effect (*F* (2, 172) = 728.21; *p* < .001), which indicates a progressive increase in RT when increasing task complexity (*p* < .001 in all cases). A significant task by group interaction (*F* (4, 392) = 1.14; *p* = .338 ) revealed that the differences between groups seem to remain fairly constant in the first three tasks decreasing in CRT and CRT‐search tasks (FT task *p* < .001, SRT *p* < .001, SRT‐SART *p* < .001, CRT *p* = .023, CRT‐Search *p* = .035). (Figure 1).

Comparisons between patients with PD and healthy controls showed that, in the FT task patients with PD exhibited increased response times (t (65) = ‐ 3.51; *p* = .001, d = 0.120).

ANCOVA, designed to measure the perceptual component and sustained alert, revealed that this was the only component where patients with PD have slower responses than controls (*F* (1, 98) = 24.63; *p* < .001, *η* ^2^ part = 0.201).

**FIGURE 2:**
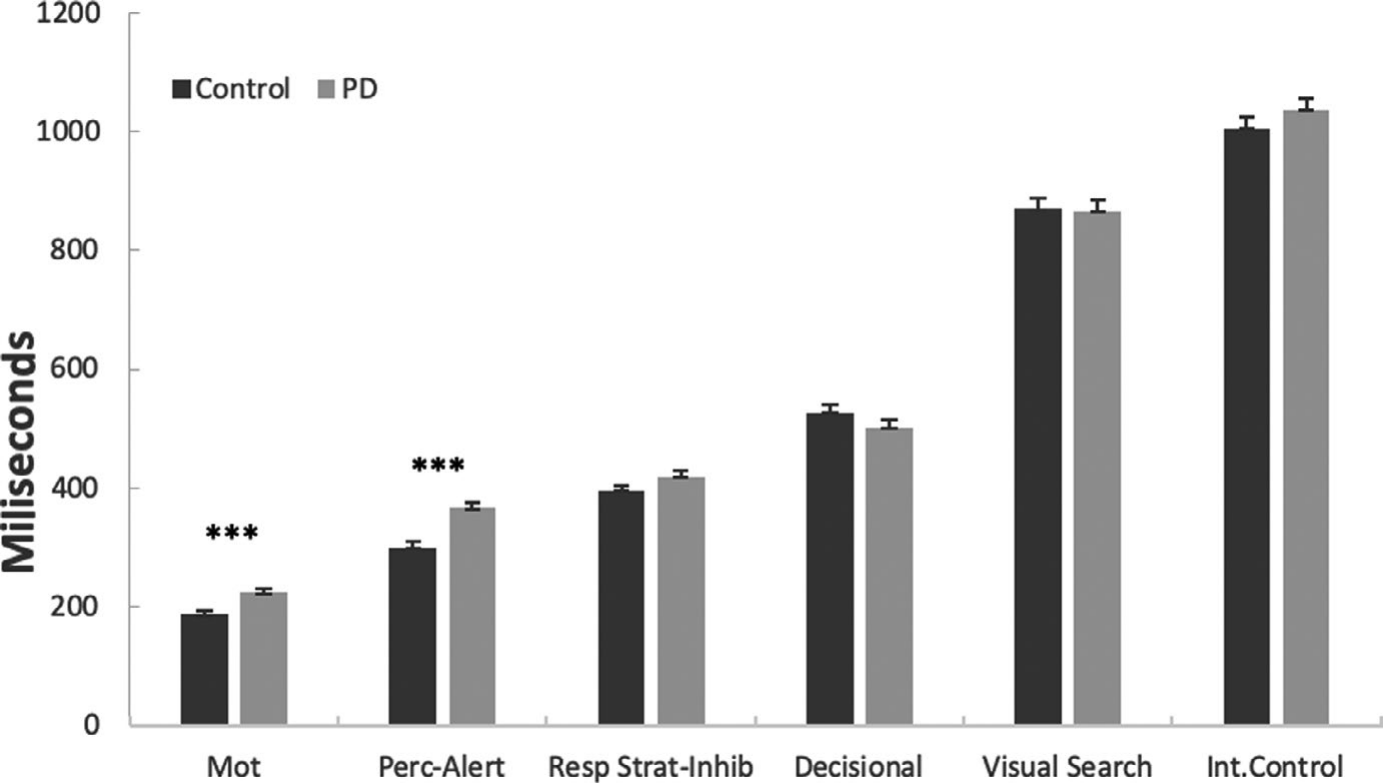
Comparisons between Parkinson's disease patients and healthy controls in different components of the stimulus‐response pathway (motor, perceptual‐alertness, response strategy‐inhibition, decisional, visual search, and interference control). Asterisks indicate statistically significant differences between groups. Mot = motor; Perc‐Alert = perceptual‐alertness; Resp Strat‐Inhib = response strategy‐inhibition; Int‐control = interference control

On the other hand, there were no differences between both groups in the rest of analysis including: 1) Response strategy‐Inhibition component measured by the SRT‐SART task using the SRT task as a covariate (*F* (1, 97) = 2.96; *p* = .089, *η^2^* part= 0.030) 2) the Decisional component measured by the CRT task using the SRT task as a covariate (*F* (1, 98) = 1.73; *p* = .191, *η^2^* part = 0.017)) 3) Visual search component, measured by the non‐target‐low interference condition, using as a covariate the target‐low interference condition of the CRT‐Search task (*F* (1, 98) = 0.02; *p* = .883, *η^2^* part= 0.000) , 4) Control of interference between both groups (*F* (1, 98) = 1.38; *p* = .243, *η^2^* part=0.014) (Figure 2).

### 3.3 Accuracy

All RT tasks were performed very efficiently, exceeding 89% of correct answers in all the tests in both groups (Table 2).

When ANOVAs with the correct answers were performed, there is a main effect of the task (*F* (2, 211) = 27.19; *p* < .001). Post hoc analysis revealed that the percentage of correct answers was higher for the SRT than for the CRT task (*p* < .001) and for the CRT‐Search task (*p* = .005). Greater for CRT‐Search than for the CRT task (*p* < .001), and higher for the TRS‐SART than for the CRT task and CRT‐Search (*p* < .001).

Group effect was not significant (*F* (1, 96) = 2.51; *p* = .116). Group interaction by percentage of correct answers in each task was not significant (*F* (3, 288) = 0.38; *p* = .766).

### 3.4 Correlation

Dopamine doses was not significantly correlated with FT (r = ‐ 0.101; *p* = .494), SRT (r = 0.270; *p* = .063), SRT‐SART (r = 0.118; *p* = .430), CRT (r = 0.093; *p* = .531), and CRT‐Search (r = 0.017; *p* = .911).

HY was not significantly correlated with FT (r_s_ = 0.143; *p* = .339), SRT (r_s_ = 0.252; *p* = .087), SRT‐SART (r_s_ = 0.252; *p* = .091), CRT (r_s_ = 0.173; *p* = .245), CRT‐Search (r_s_ = 0.203; *p* = .171).

## 4 DISCUSSION

The first objective of this study was to investigate if components of information processing (motor, perceptual, and cognitive) are globally affected in PD or, in contrast, if those components are affected in a specific manner. The results reveal a deceleration in the processing of information in PD evident in all the RTs tasks being used. This coincides with previously published data. Both SRT (Kojovic et al., 2014; Moisello et al., 2011) and CRT have been reported as slower been reported to be slower in PD than controls (Jahanshahi et al., 1992)

As expected, as task complexity increased, the RTs required in each task was longer in both groups, but surprisingly, such a **“**complexity effect” was not greater for patients with PD than for healthy control participants. In other words, the difference in performance seemed to be established in the simplest stages of processing, but it remained without major increases in more complex tasks beyond SRT. Consequently, the lack of a disproportionate complexity effect in patients with PD lead us to the suspicion of impairments focused on basic components of information processing as evidenced in the next phase of the analysis of results.

Although previous studies sing Go no‐Go task paradigm have demonstrated that patients with PD are slower and make more errors than controls (Buccino et al., 2018; Yang et al., 2018), in our study all RT tasks were performed very efficiently. Differences between groups in accuracy where not significant except for the CRT‐S task, this finding cannot be interpreted accurately as the total dopamine dosing is not standard in all the included PD subjects. Accuracy itself was high, with mean percentages of correct responses varying between 88% and 97%.

The second objective of this study was to identify which motor, perceptual and sustained alert, and cognitive components (response strategy‐inhibition, visual search, decision making, and interference control) are mediating the SIP. Of additional interest is the innovative strategy used to achieve this objective that supposes the analysis of components through ANCOVAs. The advantage of using this strategy is that no a priori assumptions are made about the functional architecture of the cognitive system (neither serial nor parallel), since ANCOVAs only estimates the amount of variance shared between the dependent variable and the covariate. As expected, the simple motor task, requiring a self‐paced tapping of a simple key as fast as possible, did show differences between patients with PD and controls according to the bradykinesia usually described in these subjects (Erro and Stamelou, 2017). Further results of this analysis unexpectedly show that perceptual and sustained alert component was the only one where patients with PD showed worse performance than controls, with no differences between both groups in the remaining components. This result suggests that motor, perceptual, and basic attentional (alertness) factors could explain by themselves most of the slowness observed in both the SRT and CRT tasks, and may explain bradyphrenia in PD. Moreover, the present results confirm conclusions from early works suggesting a lack of impairment in certain cognitive stages of information processing by using a fine grain novel methodology that allowed to isolate specific cognitive components (Smith et al., 1998; Phillips et al., 1999). This is one of the key findings of our study. In this regard, alertness has been found to be impaired from the early clinical stages of PD (Dujardin et al., 2013; Dunet et al.,; Herman et al., 2014). Preceding neuroimaging studies using SPECT have associated RTs slowness to nigrostriatal degeneration, and decreased glucose metabolism in the prefrontal cortex (Frings et al., 2020). In PD, changes are observed in multiple pathways involving various brain regions, mainly basal ganglia, thalamus, and prefrontal cortex (Wang et al., 2020). In this regard, the present results suggest that the basic nature of alertness may be important enough to justify deficits in the performance of more complex tasks, and could be conditioned to connectivity problems between the frontal lobe and the basal ganglia (Dirnberger and Jahanshahi, 2013; Szewczyk‐Krolikowski et al., 2014). The clinical impact of these results are in line with findings suggesting that the balancing effects of deep brain stimulation (DBS) in pedunculopontine nucleus of patients with PD would be mediated by increases in general alertness and attentional functions (Thevathasan and Moro, 2019).

These findings are of crucial importance for addressing both the early detection of cognitive disorders associated with Parkinson's disease, the rehabilitation strategies implemented to treat them, and the influence of cognitive impairment on motor symptoms such as balance. The delimitation of the altered processing components confirms that the affected processes are at the lowest level of cognitive processing which is consistent with neuroimaging data that point to alterations in the salience network in Parkinson's disease (Putcha et al., 2016). It is worth mentioning that the salience network is involved in detecting and filtering salient stimuli, and the relative salience of these inputs determines which are more likely to be cortically processed. This network is involved in a variety of complex functions, including communication, social behavior, and self‐awareness through the integration of sensory, emotional, and cognitive information (Toga, 2015). Salience network activity would have a fundamental role in detecting and reacting to stimuli capable of engaging one´s attentional and motivational status, regardless of the sensory channel. Furthermore, there has been described a close relationship between the salience network and the mesolimbic dopamine system (McCutcheon et al., 2019). Cognitive effects of the dopaminergic medication have been targeted to its influence in the salience network (Nagy et al., 2012). Although there is no agreement in the effect of dopaminergic treatment on reaction times, it has a clear influence on some cognitive processes. Normalization of impulsivity and inhibition process‐related reaction times have been reported with the use of dopamine (Yang et al., 2018) and on the other hand, impulse control disorder is associated with faster reaction times with a higher proportion of errors in dopamine agonist treated patients (Djamshidian et al., 2014). All included patients in our study were in "ON state" during the measures and those using agonist drugs did not report impulse control disorders related symptoms. The total dopamine dose and HY stage did not show a correlation with RTs. Although bradyphrenia plays a major role in OFF state, our patients were examined in ON state in order to avoid the impact of other motor (bradykinesia) and nonmotor symptoms such as fatigue and pain on the cognitive performance besides bradyphrenia. Although we did not find a relationship between the RTs and the dose of dopamine, the heterogeneity of the PD group with respect to the total equivalent dose of levodopa could be a limitation, since dopaminergic treatment could have both positive and negative effects on cognition (Cools, 2006; Cools et al., 2001). Although all the evaluations were performed in ON state, there may have been uncontrolled differential effects of medication depending on the phase of the disease.

## 5 CONCLUSIONS

Our results indicate that although PD patient's performance was slow on all RT tasks, such a slowness could not be attributed to a generalized deficit in all the components of information processing being involved. Particularly, the present component analysis using a series of ANCOVAs suggested that the level of cognitive deceleration in PD was determined by a deterioration focused on mainly perceptual and motor components and independently of the task complexity. In addition, the present study suggests that alterations in the brain networks involved in alertness to react to stimuli could justify the slowness of information processing in Parkinson´s disease. Replicating the observed effect sizes in future, PD researches will become interesting to verify how relevant each component is accounting for slowness of information processing. Our results could be of great value for designing new neurorehabilitation approaches targeted to attention processes for the treatment of bradyphrenia in PD.

## CONFLICTS OF INTEREST

The authors declare no competing financial interests.

## AUTHOR CONTRIBUTION

Conceptualization, A.A. and J.R.; methodology, A.A. and J.R.; software, M.R., J.P., and G.L.; formal analysis, A.A., J.P., M.R.L., G.L.; investigation, A.A. and J.R.; resources, J.R.; data curation, A.A., J.A.; writing—original draft preparation, J.A., A.A., and J.R.; writing—review and editing, A.A., M.R., J.P., G.L., J.B.L., E.L., and J.R.; visualization, A.A.; supervision, J.R.; project administration, J.R.; funding acquisition, J.R. All authors have read and agreed to the published version of the manuscript.

### Peer Review

The peer review history for this article is available at https://publons.com/publon/10.1002/brb3.2031.

## Data Availability

The data that support the findings of this study are available from the corresponding author upon reasonable request.

## APPENDIX A Comparison between patients with PD and healthy controls

**Table:**

	Linearity		Homoscedasticity		Hom Reg
	Controls *r* (sig)	PD *r* (sig)	Levene DV Sig	Levene Cov Sig	Sig
SRT cov FT	250 (071)	151(304)	288	001	308
SRT‐SART cov SRT	555 (000)	586 (000)	059	288	438
CRT cov SRT	,526 (,000)	702 (000)	622	288	466
*No‐Target‐High Int*. cov *No‐Target‐Low Int.**	840 (000)	852 (000)	006	028	0.295
*No‐Target‐Low Int*. cov *Target‐Low Int.**	855 (000)	899 (000)	028	001	173

Abbreviations: Conditions of the CRT‐Search task. Hom Reg: homogeneity of regression; CRT: Choice Reaction Time; FT: Finger tapping, SRT: Simple Reaction Time; Int: Interference; SRT‐SART: Simple Reaction Time–Sustained Attention to Response Task.
